# Alternative Anticoagulation for Patients with Heparin-Induced Thrombocytopenia on ECMO: A Narrative Review

**DOI:** 10.3390/biomedicines13112705

**Published:** 2025-11-04

**Authors:** Dragana Unic-Stojanovic, Petar Vukovic, Ivan Ilic, Milica Miljkovic Stojicic, Slobodan Tanaskovic, Nikolina Kangrga, Sasa Rajsic

**Affiliations:** 1 Faculty of Medicine, University of Belgrade, 11000 Belgrade, Serbiaivan1ilic@yahoo.com (I.I.);; 2 Institute for Cardiovascular Diseases Dedinje, 11000 Belgrade, Serbia; 3 Department of Anaesthesia and Intensive Care Medicine, Medical University of Innsbruck, 6020 Innsbruck, Austria

**Keywords:** heparin-induced thrombocytopenia, extracorporeal membrane oxygenation, anticoagulation monitoring, thrombosis, bleeding, cardiac surgery, critical care

## Abstract

Extracorporeal membrane oxygenation (ECMO) is a continuously evolving and increasingly utilized life-support therapy. ECMO requires systemic anticoagulation, which exposes patients to an increased risk of heparin-induced thrombocytopenia (HIT). Clinical experience with alternative anticoagulants in this setting remains limited. The 2022 Extracorporeal Life Support Organization (ELSO)—Anticoagulation Guidelines provide no specific recommendations regarding anticoagulant selection for ECMO patients with HIT. This article aims to review current practices, available evidence, and most recent advances concerning the use of alternative anticoagulants in ECMO patients with HIT. In patients with a high suspicion or confirmed diagnosis of HIT, management includes discontinuing all forms of heparin exposure and initiating an alternative anticoagulant, such as a direct thrombin inhibitor and/or factor Xa inhibitor. Direct thrombin inhibitors act independently of antithrombin and have a short half-life, providing a more consistent and predictable anticoagulation effect. Most available data, primarily from retrospective studies, describe the use of argatroban in ECMO patients with HIT. Bivalirudin has also been used as an alternative anticoagulant in this population, with no significant increase in bleeding or thrombotic complications. However, the current evidence remains limited to small, retrospective, single-center or case–control studies. Fondaparinux has shown effectiveness in the HIT setting and appears to have a low risk of complications. Factor XIIa inhibitors represent a novel class of anticoagulants currently under investigation, evaluated only in animal models. Growing clinical experience with alternative anticoagulants, particularly direct thrombin inhibitors, suggests that their use will likely become a primary focus in ECMO anticoagulation management in the coming years.

## 1. Introduction

Extracorporeal membrane oxygenation (ECMO) is a form of mechanical circulatory support used in patients with severe respiratory or cardiac failure. ECMO should be considered in cases of acute, potentially reversible respiratory failure and in severe cardiogenic shock refractory to conventional management. In recent years, the indications for ECMO have expanded to include various clinical scenarios, including its use during cardiopulmonary resuscitation (eCPR), as a bridge to lung or heart transplantation, for rewarming in accidental deep hypothermia, and for resuscitation following severe trauma [1]. 

ECMO can be established as a venovenous (VV-ECMO), primarily used for respiratory failure, and venoarterial (VA-ECMO), which supports patients with cardiac failure. The main principle of ECMO involves draining blood from large vessels into an oxygenator, where venous blood is enriched with oxygen and carbon dioxide is removed. The oxygenated blood is then returned to either the venous (VV-ECMO) or arterial (VA-ECMO) circulation using a centrifugal pump. 

Contact between blood and the artificial surfaces of the ECMO circuit introduces a significant risk of circuit thrombosis, requiring systemic anticoagulation. Exposure of blood to non-endothelial materials, such as the tubing and oxygenator, activates complex inflammatory and coagulation pathways. As a result ECMO disturbs hemostatic balance and homeostasis [2]. Inflammation triggers coagulation activation, impairs natural anticoagulant mechanisms, and disrupts the fibrinolytic system. The ensuing hyperinflammation elevates acute-phase reactant proteins and stimulates various immune pathways. Persistent activation of these systems leads to platelet and coagulation factor consumption, increasing the risk of severe bleeding. This phenomenon is described as “slow” disseminated intravascular coagulation (DIC).

Bleeding complications in patients receiving ECMO can be classified as minor, such as epistaxis, surgical wound bleeding, cannulation site bleeding, or gastrointestinal bleeding, and major, life-threatening, such as intracranial hemorrhage, pericardial tamponade, pulmonary, or retroperitoneal bleeding. The overall incidence of bleeding has been reported to be as high as 49% [3], whereas the incidence of intracranial hemorrhage or infarction is about 8% [4].

Multiple studies have attempted to identify risk factors associated with bleeding during ECMO and found that higher SAPS III (Simplified Acute Physiology Score) score, longer ECMO duration, prolonged aPTT, post-surgical ECMO, low inflammatory response, hemoglobin < 9 g/dL, fibrinogen < 2 g/L, pH < 7.12, and body mass index below 25 kg/m^2^ are associated with an increased risk of bleeding [5,6,7]. Patients who experience bleeding have longer ICU and hospital stays, and higher mortality rates [5]. In contrast, thromboembolic complications may range from deep vein thrombosis to acute thrombosis of the oxygenator or circuit tubing, which may lead to system failure, one of the most critical emergencies during ECMO [8].

To prevent and manage thromboembolic events, systemic anticoagulation remains the standard of care. The most commonly used anticoagulant in ECMO patients is unfractionated heparin (UFH). It is a mixture of glycosaminoglycans of different molecular weights that acts primarily by binding to antithrombin (AT) to form a complex that inhibits thrombin and factor Xa [9]. Although the therapeutic goal is to achieve a stable plasma heparin concentration, the relationship between heparin dosage and effect is non-linear [10]. 

UFH offers several advantages, including a well-established mechanism of action, low cost, ease of titration, rapid onset, and reversibility with protamine [11]. Moreover, anticoagulation can be monitored with point-of-care devices, such as activated clotting time (ACT), or through laboratory tests such as activated partial thromboplastin clotting time (aPTT), anti-factor Xa activity, or viscoelastic testing. For these reasons, current guidelines continue to recommend UFH as the first-line anticoagulant during ECMO support [12].

During states of hyperinflammation, the anticoagulant efficacy of UFH may be diminished due to increased binding of heparin to acute-phase proteins, such as factor VIII and fibrinogen, as well as decreased plasma concentrations of AT [5]. A key limitation of heparin therapy lies in its nonspecific binding to multiple targets, including AT, endothelial cells, circulating plasma proteins, and macrophages. These interactions alter its pharmacokinetic properties and dose–response relationship, contributing to complications such as heparin resistance and heparin-induced thrombocytopenia (HIT) [12].

## 2. Heparin-Induced Thrombocytopenia—HIT

### 2.1. Types and Incidence

To date, the understanding of HIT in patients receiving ECMO remains limited. Most studies reporting HIT in this context are constrained by small sample sizes, single-center designs, and retrospective nature. As a result, the literature is inconsistent, particularly regarding the prevalence and mortality rates of HIT among ECMO patients [13,14]. Given the increasing use of ECMO, this topic is of growing clinical importance, and updated systematic data on HIT in this population are needed.

**Figure 1:**
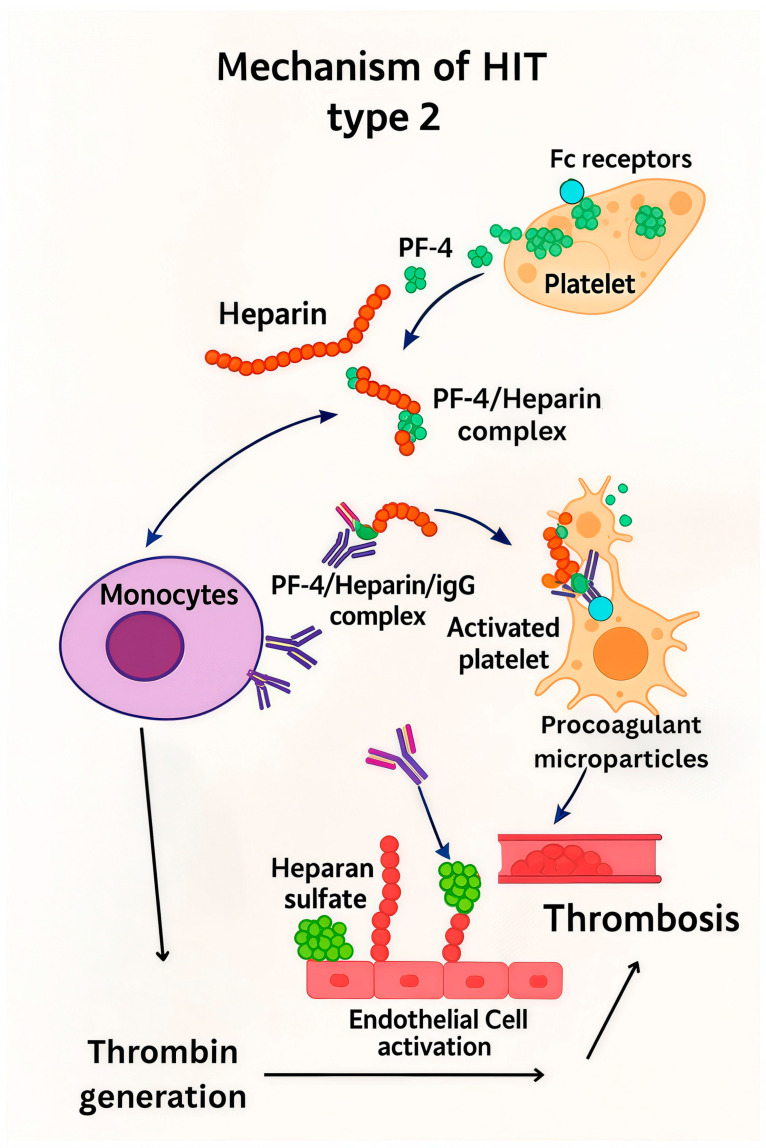
Schematic illustration of heparin-induced thrombocytopenia (HIT): IgG antibodies bind to platelet factor 4 (PF4)–heparin complexes, resulting in cross-linking of FcγRIIA receptors on platelets and monocytes, which triggers their activation and promotes a prothrombotic state.

Heparin can induce two distinct types of thrombocytopenia. Type I HIT, a non-immune form, typically develops within the first few days of heparin therapy and is more common than type II. In contrast, type II HIT is an immune-mediated complication of anticoagulant therapy that can occur with both UFH and low-molecular-weight heparin [15]. Type II HIT is characterized by a >50% reduction in platelet count from the post-heparin peak value [15], typically developing 5 to 10 days after heparin initiation. Key pathological features include the formation of heparin-dependent, platelet-activating IgG antibodies, which generate a state of hypercoagulability [16]. These IgG antibodies bind to platelet factor 4 (PF4)-heparin complexes, leading to intravascular activation through FcyRIIA receptor cross-linking (Figure 1). This process induces a prothrombotic and hypercoagulable state, ultimately resulting in thrombocytopenia [17]. 

The reported incidence of Type II HIT in adults ranges from 0.2% to 5% [16,18], although available data are inconsistent, suggesting rates from <0.36% to 17% [19,20,21,22]. A retrospective analysis of 118 ECMO patients reported a prevalence of HIT and HITT (Heparin-Induced Thrombocytopenia with Thrombosis) of 8.3% and 7.3%, respectively [14], with complications occurring in approximately 25% to 50% of affected patients [23,24]. Notably, HITT in ECMO patients has been associated with a mortality rate approaching 50%. Compared with HIT-negative patients, those testing positive for HIT typically require longer ECMO support, are younger, exhibit higher HIT ELISA optical densities, and reach their platelet count nadir later during therapy [14]. A recent meta-analysis of 21 studies reported thrombocytopenia rates ranging from less than 1% to 22% among patients receiving VA-ECMO [25], with a pooled prevalence of confirmed HIT cases of 3.7%. The wide variability in reported HIT incidence likely reflects the heterogeneity in patient populations, differences in anticoagulation protocols and variations in HIT screening methods. Consequently, the elevated risk of HIT observed in ECMO patients may not directly result from arterial cannulation or ECMO-related vascular and inflammatory injury [26]. Instead, it may reflect prior and prolonged exposure to UFH before ECMO initiation [26]. 

Thrombocytopenia during ECMO is multifactorial, encompassing factors such as cardiopulmonary bypass (CPB), consumptive coagulopathy, blood drainage, sepsis, multi-organ failure, platelet consumption, hemodilution, and bleeding [26]. Following the cessation of heparin therapy, patients with confirmed HIT demonstrate a faster recovery in platelet count (≥50% increase from nadir) compared with HIT-negative thrombocytopenic patients, indicating differing pathophysiological mechanisms [27]. However, in ECMO patients with HIT, platelet recovery is delayed compared with those supported by CPB alone, likely due to circuit-related platelet activation and other concurrent causes of thrombocytopenia [27].

### 2.2. HIT Diagnostic

**Figure 2:**
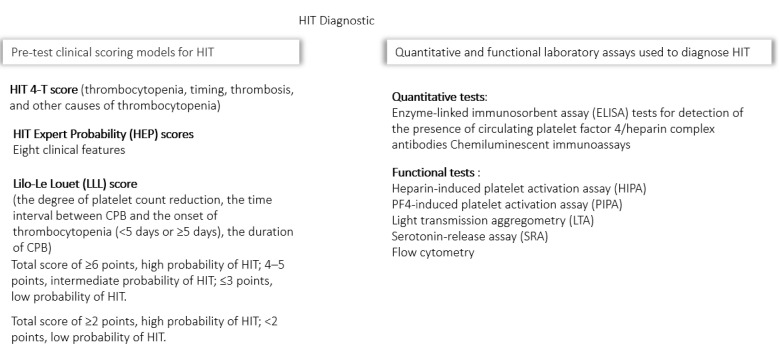
Heparin-induced thrombocytopenia (HIT) diagnostics. HIT Expert Probability (HEP) score; LLL, Lilo-Le Louet score; CPB, cardiopulmonary bypass; ELISA, enzyme-linked immunosorbent assay; HIPA, heparin-induced platelet aggregation; PIPA, PF4-induced platelet activation assay; LTA, light transmission aggregometry; SRA, serotonin release assay.

The diagnosis of HIT in ECMO patients is particularly challenging, and both underdiagnosis and overdiagnosis can lead to potential problems [28]. Failure to identify HIT increases the risk of venous and/or arterial thrombosis, limb amputation, or death. Continuing UFH therapy in affected patients may exacerbate thrombotic complications; if heparin is not discontinued and replaced with an alternative anticoagulant, the risk of thrombosis is estimated at 40–50%, especially in patients with repeated heparin exposure [15,28,29]. Conversely, unnecessary discontinuation of heparin due to a misdiagnosis may increase bleeding risk when alternative anticoagulants are used to treat thrombocytopenia and may also increase hospital costs. The difficulty of diagnosing HIT during ECMO is further complicated by the fact that sepsis and DIC can present with similar clinical features (Figure 2).

The 4-T score for HIT, which considers thrombocytopenia, timing, thrombosis, and other causes of thrombocytopenia, is commonly used to assess the pretest probability of HIT [24]. However, it has limited sensitivity and specificity and has not been extensively validated or designed for use in the context of mechanical circulatory support (MCS). While the score may be numerically higher in patients with confirmed HIT, this difference is not statistically significant [16]. The pretest probability is categorized as low (0–3 points), intermediate (4–5 points), or high (6–8 points). For patients with a low pretest probability, further laboratory testing is not recommended, except in selected ECMO patients (BSH: Grade 2C) [30]. Kram et al. demonstrated that the predictive accuracy of both the 4-Ts and HIT Expert Probability (HEP) scores was limited in patients supported with various MCS devices, and that low scores were insufficient to reliably rule out HIT [31]. In patients with intermediate or high pretest probability, laboratory testing is warranted. Additionally, platelet count trends alone do not reliably distinguish between confirmed and excluded HIT [16,19,26]. Suspicion should increase if a patient’s platelet count declines during ongoing heparin therapy. Given the frequent occurrence of thrombocytopenia due to bleeding, surgery, or infection in MCS patients, both the 4-Ts and HEP scores may overestimate the likelihood of HIT in these patients.

The Lilo-Le Louet (LLL) score is widely used for the diagnosis of HIT with a 97% negative predictive value in patients following CPB [32]. This score is specifically developed for cardiac surgery patients. It is based on three factors: (1) the degree of platelet count reduction (categorized as pattern A or B), (2) the interval between CPB and onset of thrombocytopenia (<5 days or ≥5 days), and (3) CPB duration. Pattern A is characterized by a transient post-CBP platelet recovery, followed by a secondary drop after more than 4 days, and is more predictive of HIT than Pattern B, which involves a persistent thrombocytopenia immediately after CPB without recovery [32,33,34]. This approach may provide better predictive accuracy compared with the 4Ts or HEP scores, especially in MCS patients [31].

The enzyme-linked immunosorbent assay (ELISA) for detection of platelet factor 4 (PF4)-heparin complex antibodies in plasma or serum is widely available, rapid, and very sensitive but lacks specificity in the ECMO setting [35]. Several studies have established the ELISA optical density (OD) thresholds in the general population [36,37]. Manufacturers typically define a positive result as an OD >0.4 units; however, this threshold demonstrates limited specificity and may reduce the clinical relevance of test results [37]. 

Functional tests are confirmatory functional assay with higher specificity and are indicated to confirm the diagnosis. These include C-serotonin release assay (C-SRA), heparin-induced platelet activation (HIPA) test, heparin-induced platelet aggregometry (measures aggregation), lumiaggregometry, and flow cytometry. Rapid initiation of functional assays is recommended for all patients presenting with a positive antibody screening results in conjunction with strong clinical likelihood of HIT. Although the SRA and HIPA tests are more specific, these tests are usually performed at reference laboratories and may take 5 to 7 days to process, reducing their immediate utility for clinical decision making.

The diagnosis of HIT during VA-ECMO therapy has been associated with longer ICU stays but does not appear to affect overall survival [13,16,20].

## 3. Alternative Anticoagulants

**Figure 3:**
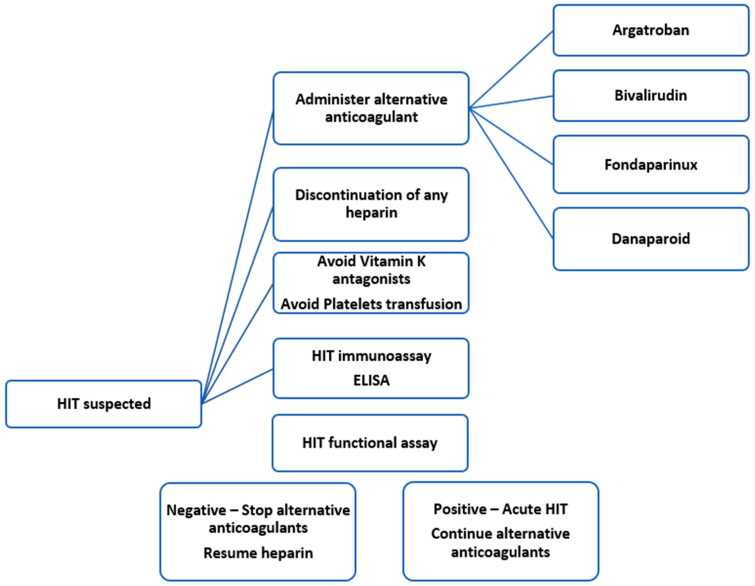
Management steps for heparin-induced thrombocytopenia (HIT) complicating extracorporeal membrane oxygenation support. ELISA, enzyme-linked immunosorbent assay; HIPA, Heparin-Induced Platelet Aggregation; PIPA, PF4-induced platelet activation assay; LTA, light transmission aggregometry; SRA, serotonin release assay.

In patients with a high clinical suspicion or confirmed HIT diagnosis, management involves immediate discontinuation of all forms of heparin exposure, including heparin-containing flush solutions. An alternative anticoagulant, such as a direct thrombin inhibitor (DTI) or a factor Xa inhibitor, should then be initiated [1,38] (Figure 3). Delaying treatment with an alternative anticoagulant is associated with a daily risk increase of 5–10% for thrombotic events, limb loss, or death [39].

The optimal anticoagulation strategy for ECMO patients who developed HIT remains uncertain. Current guidelines, including those from the American College of Chest Physicians and the ELSO Guidelines, do not provide specific recommendations for this patient group [12,40,41]. Bivalirudin is recommended for patients with acute HIT who require urgent cardiac surgery, rather than heparin or other non-heparin anticoagulants. For patients with HIT undergoing acute percutaneous coronary intervention, the use of antiplatelet agents (grade 2C) in combination with either bivalirudin (grade 2B) or argatroban (grade 2C) is advised (Table 1).

Direct thrombin inhibitors (DTIs) are anticoagulants with a short half-life that act independently of AT by directly binding to thrombin. They provide a more consistent and predictable anticoagulant effect in situations where AT activity is low or variable and demonstrate more reliable pharmacokinetics, as well as greater efficiency in reducing thrombin generation compared with UFH [12,38]. Unlike heparin, DTIs do not bind to plasma proteins or blood cells, making them less susceptible to fluctuations in serum chemistry or cell counts. This characteristic allows for more predictable dosing and a consistent anticoagulant effect, potentially reducing bleeding risk. DTIs are not associated with immune-mediated thrombocytopenia, including HIT. Additionally, DTIs inhibit both thrombin and thrombin bound within clots, enhancing their overall anticoagulant effectiveness [39]. Among synthetic DTIs, argatroban, bivalirudin, and lepirudin have been used in CPB, ECMO, and VAD support; however, lepirudin is now rarely available.

### 3.1. Argatroban

#### 3.1.1. Mechanism of Action

Argatroban is a synthetic L-arginine derivative with concentration-dependent anticoagulant activity. Its onset of action occurs within 30 min, and its half-life is approximately 45 min. Argatroban is primarily metabolized in the liver via hydroxylation and aromatization of the 3-methyltetrahydroquinoline ring. During extracorporeal circulation, its low distribution volume (180 mL/kg) is increased, resulting in limited tissue diffusion and distribution mainly within extracellular space. Argatroban binds univalently to the active site of thrombin, in contrast to bivalirudin, which binds bivalently to both the active site and exosite-1 on thrombin [42] (Table 2). The antidote for argatroban still does not exist. It is considered a safe anticoagulation option for patients with HIT and renal failure, especially in the ICU setting [43]. However, argatroban is contraindicated in patients with severe hepatic impairment (Child-Pugh Class C) due to the high risk of drug accumulation [42].

#### 3.1.2. Dosing and Monitoring of Argatroban

Argatroban therapy does not require a loading dose. In patients on ECMO, the initial infusion rate should range between 0.2 and 2 mcg/kg/min, depending on the target activated partial thromboplastin time (aPTT) value [43]. According to Rajsic et al., argatroban dosing should be approached with caution, particularly in patients with hepatic impairment, where maintenance dose as low as 0.1–0.2 mcg/kg/min may be necessary [1]. 

Monitoring the effect of argatroban can be performed using several assays, including ACT, aPTT, thrombin time, diluted thrombin time (dTT), ecarin clotting time (ECT), viscoelastic methods, chromogenic antithrombin activity (CAA) or measuring plasma concentrations [44]. aPTT should be measured two hours after starting the infusion to prevent excessive anticoagulation and minimize bleeding risk [42]. However, there are significant limitations to using aPTT for argatroban monitoring. These include variability related to the method and reagents used, intra- and inter-patient variability, and potential interference from lupus anticoagulant, elevated factor VIII, fibrinogen, vWF, and CRP levels, as well as reduced coagulation factors production (especially FXII). At higher DTI concentrations, aPTT may underestimate the true anticoagulant effect [44]. 

More precise monitoring can be achieved using tests like the diluted thrombin time (TT), which is correlated with a drug level nomogram, or the ecarin clotting time (ECT) [12,44]. However, these alternative tests have not been widely validated or approved by regulatory authorities in most countries [42]. Measurement of plasma argatroban concentration is considered the most accurate method. Swiss guidelines recommend a target range of 0.4–1.5 μg/mL [45], while French guidelines recommend a target range of 0.25–1.5 μg/mL, particularly in patients with prolonged baseline aPTT prior to argatroban initiation [46]. Unfortunately, such assays are not readily available in most laboratories. Argatroban, unlike other alternative anticoagulants, elevates the international normalized ratio (INR). During transition to warfarin, which should only be initiated once platelet count has normalized, clinicians typically overlap both agents (a non-heparin anticoagulant and a vitamin K (VKA) antagonist therapy) for 4 to 5 days, maintaining an INR > 4.0.

#### 3.1.3. Clinical Studies

Numerous studies, most of them retrospective, have investigated the use of argatroban in ECMO patients diagnosed with HIT [13,16,21,27,28,47,48,49] (Table 3). Reported HIT prevalence ranges 3.2% to 6.4%, with no significant difference between ECMO configurations (VA vs. VV ECMO *p* = 0.47) [27].

No difference in in-hospital mortality was observed between patients with and without HIT (43% in HIT vs. 38% non-HIT patients, *p* > 0.999) [16] (31.6% in HIT vs. 32.2% in non-HIT patients, *p* = 0.79) [27]. However, Glick et al. reported significantly higher in-hospital mortality among patients with suspected HIT (14/23, 61% patients in HIT group vs. 31/96 patients without HIT, 32%; *p* = 0.01), likely reflecting more severe thrombocytopenia and a generally sicker patient population [21]. Arachchillage et al. similarly found no difference in mortality rates in patients on ECMO with HIT (1/2, 50%) and those with HIT and thrombosis [5/17, 29.4%]; *p* = 0.96) [27]. Neurological outcomes at hospital discharge and at one month (35% vs. 38%, *p* > 0.999), as well as mortality rates at three and twelve months, were also comparable between patients with excluded and confirmed HIT [16].

Bleeding remains the most common complication during ECMO therapy. Some studies found no difference in bleeding incidence between HIT and non-HIT patients [16], whereas others reported a higher incidence of bleeding in patients without HIT [13,27]. Lubnow et al. reported an increased bleeding rate in HIT patients [47]. Several studies did not report bleeding outcomes [21,28,50].

The incidence of thrombosis varied across the studies. While some authors reported no difference between groups [16], others reported a higher rate of thrombotic events in HIT patients [27,47]. Several studies did not provide the data on thrombosis incidence [21,28,50].

Argatroban therapy was monitored with aPTT, though different anticoagulation goals are reported across the studies. These targets varied from 50 s for suspected HIT and 60 s for confirmed HIT [28,47], to 78 s in the study conducted by Archchallange et al. 2020 [48].

Although switching from heparin to an alternative anticoagulant such as argatroban may initially increase drug costs, when accounting for the costs associated with HIT testing, monitoring, and potential complications, the overall treatment costs are comparable [47,50].

While there are no direct comparative studies assessing argatroban efficacy in HIT/HITT patients versus those with thrombocytopenia of other etiologies, treatment of HITT with argatroban has been associated with a significantly faster platelet count recovery compared to historical control populations [51]. Pabst et al. reported that after discontinuation of systemic heparin and switching to argatroban in patients with HIT, platelet counts increased from a mean of 59.8 k/μL at the time of HIT diagnosis to a mean of 280.2 k/μL at 14 days post-heparin discontinuation, even while maintaining a heparin-bonded circuit [28]. Eleven out of the fourteen HIT patients (78.6%) survived to discharge. Causes of death were multiorgan failure and intracranial hemorrhage. HIT may have played a role in these outcomes, as it is known that HIT increases the risk of thrombotic events that can lead to stroke, brain bleeding, and multiorgan failure. The time to achieve a ≥50% increase in platelet count from the nadir was significantly reduced in patients with confirmed HIT treated with argatroban compared to those without HIT, indicating different pathophysiological mechanisms of thrombocytopenia [27]. However, HIT patients on ECMO required longer to reach a >50% platelet recovery than CPB patients, likely due to ECMO circuit-related effects and other thrombocytopenia-inducing factors [27].

In 2018, Kimmoun et al. [13] reported a retrospective study including patients on VA-ECMO from 20 French centers between 2012 and 2016 [13]. Of 5797 screened patients, 39 were hospitalized for more than 3 days with a high clinical suspicion of HIT and positive anti-PF4/heparin antibodies. Alternative anticoagulation therapy consisted primarily of argatroban in 11 of 21 confirmed HIT patients (52.4%) and danaparoid in 10 of 21 patients (47.6%). While an increase in platelet count following the change in anticoagulation therapy was expected in confirmed HIT, a similar rise in platelet count was also observed in patients excluded from the HIT diagnosis. One possible explanation is that over 50% of patients in the excluded HIT group were successfully weaned from VA-ECMO after 7 days, which was associated with platelet recovery [13]. Alternative causes of thrombocytopenia, such as sepsis or drug-induced thrombocytopenia, might have played a role in the platelet recovery.

Mang et al. described patients with coronavirus disease 2019 (COVID-19) requiring V-V ECMO with rapid deterioration of oxygenator function, requiring multiple administrations of rtPA. After heparin was replaced with argatroban, oxygenator performance stabilized [52]. Among forty-one COVID-19 patients requiring VV ECMO, seven (17%) tested positive for HIT by ELISA, but only one (2%) was confirmed to have HIT type II by a confirmatory HIPA test.

A retrospective single-center study reported that the use of heparin-coated circuits did not lead to a further decrease in platelet count or higher complication rates [47]. Notably, two patients on non-heparin-coated circuits did not experience faster platelet recovery after transitioning to argatroban, in contrast to those on heparin-coated circuits.

### 3.2. Bivalirudin

#### 3.2.1. Mechanism of Action

Bivalirudin inhibits plasma thrombin, clot-bound thrombin, and collagen-triggered platelet activation without requiring antithrombin III. About 80% of the drug is metabolized through proteolysis, which is nonorgan-dependent, while the remaining one-fifth is excreted unchanged via renal pathways [53,54,55,56]. This metabolism supports a predictable relationship between bivalirudin dosage and anticoagulant efficacy [53,57]. The onset of action occurs within 2–4 min, and half-life is approximately 25 min. Compared with argatroban (39–51 min) and lepirudin (78 min), bivalirudin’s shorter half-life facilitates precise titration of anticoagulation. Because of its short half-life, discontinuation of the infusion is typically sufficient to reverse anticoagulation in the event of accidental overdose or significant bleeding. In more severe cases, adjunctive measures such as plasma transfusion, administration of prothrombin complex concentrate, or hemodialysis may be required [56]. Approximately 25% of bivalirudin can be removed through hemodialysis.

#### 3.2.2. Dosing and Monitoring of Bivalirudin

Only a limited number of studies have reported the use of a loading dose of bivalirudin, ranging from 0.2 to 0.75 mg/kg [53]. Initial bivalirudin infusions rates in VV-ECMO and VA-ECMO (excluding postcardiotomy ECMO) are usually set at 0.02–0.05 μg/kg/min [58]. Assessment of renal function is essential to determine the appropriate initial dose of bivalirudin, with or without a loading bolus. Reported maintenance infusion rates vary widely, ranging from 0.05 to 1.75 mg/kg/h, with a mean rate of 0.27 ± 0.37 mg/kg/h [53]. Lopez et al. reported results from the largest retrospective cohort of HIT/HITT patients, specifically evaluating dosing strategies [59]. A median infusion rate of 0.05 mg/kg/h was sufficient to achieve the desired aPTT, representing a lower dose than those previously documented in ECMO populations [59,60,61]. Patients receiving ECMO in combination with CVVH required the same median bivalirudin dose to achieve therapeutic aPTT as those on ECMO alone [59,62]. The longest reported duration of bivalirudin usage exceeded 60 days. A retrospective analysis of HIT patients with elevated BMI demonstrated that total body weight-based dosing most reliably predicted attainment of the target aPTT [63].

The anticoagulant effect of bivalirudin can be monitored using several laboratory tests, most commonly aPTT and ACT. aPTT monitoring shows an excellent correlation with bivalirudin’s anticoagulant effect, with the same standard target range as for UFH of 45–80 s [53,58]. ACT can also be used, with target values from 160 s to 220 s, and serves as an adjunct to aPTT in some centers [53,56]. If the baseline aPTT levels are abnormal, a dTT, chromogenic anti-IIa testing and blood concentration of drug can be used [58]. Nowadays, ACT and aPTT are increasingly avoided due to their susceptibility to multiple confounding factors. More specific assays, such as drug concentration and ROTEM, may provide more reliable measurements of bivalirudin’s effect.

#### 3.2.3. Clinical Experience

Due to the limited data, standardized protocols for bivalirudin administration in ECMO patients with HIT have not been clearly established. Bivalirudin can be used either as an initial anticoagulant in ECMO or as a secondary agent in patients with HIT or UFH resistance. It has been used off-label in ECMO without significant increase bleeding or thrombosis [40,57]. However, the available evidence is limited to small, retrospective, single-institution studies or case–control studies (Table 3).

When comparing bivalirudin and UFH, similar mortality rates and thrombotic complications are reported [64]. However, bivalirudin is associated with lower bleeding rates [65,66,67,68], less variability in ACT and aPTT, which remain within the therapeutic range for longer periods, likely due to its specific mechanism of action [61,64], and a reduced need for allogeneic blood transfusions [61,69]. Despite these benefits, concerns remain regarding bivalirudin´s rapid proteolytic cleavage, which may increase the risk of thrombosis in areas of stagnant blood, particularly in a non-ejecting, unvented left ventricle (LV) [60,70]. However, a recent systematic review and meta-analysis including 10 retrospective observational studies and 847 patients suggested that bivalirudin may significantly reduce thrombotic events, in-circuit thrombosis, and in-hospital mortality [68]. Other data showed that the incidence of bleeding events with bivalirudin is comparable to that with UFH [53,60,61].

Transitioning ECMO patients with HIT to bivalirudin is associated with increased platelet counts. Zhong et al. found that the mortality in ECMO patients with HIT treated with bivalirudin was similar to that of ECMO patients without HIT who are treated with UFH [53].

Several studies that included adult ECMO patients with HIT and switched to bivalirudin reported no cases of bleeding, thrombosis, or mortality in this cohort [34,71]. According to the conclusion of authors, bivalirudin and argatroban are safe alternatives to heparin and should be initiated in patients with high suspicion for HIT while awaiting confirmatory SRA results [34,71].

Giuliano et al. reported that the composite rate of thrombotic or hemorrhagic complications was lower in ECMO patients with suspected or confirmed HIT receiving bivalirudin [72]. Less severe hemorrhage—as indicated by lower blood product transfusion—was registered in the bivalirudin group. In-hospital mortality was comparable between patients treated with bivalirudin and heparin (69%) and those treated with heparin alone (62%), despite greater illness severity in the bivalirudin group (higher rates of prior intracranial hemorrhage, longer median ECMO duration, higher SOFA scores on ECMO day one, and a greater need for continuous renal replacement therapy (CRRT)).

### 3.3. Lepirudin

Lepirudin is a recombinant form of hirudin. It inhibits thrombin in a bivalent manner, targeting the catalytic site as well as exosite-1. It has an elimination half-life of 1 to 2 h, with bolus administration achieving peak aPTT within 10 min. As lepirudin is primarily eliminated renally, dose adjustments are required in patients with acute kidney injury. Significant adverse effects include bleeding and the development of antihirudin antibodies. The bleeding risk may be mitigated by careful dose titration and regular laboratory assessment [73]. Anaphylactic reactions are rare and generally linked to bolus administration. In the absence of an antidote, the use of lepirudin remains challenging in certain clinical contexts, particularly in high-risk settings such as CPB. It has been utilized successfully in patients undergoing ECMO with contraindications for UFH, with aPTT- and ACT-guided titration of doses. In these patients, bleeding or thrombosis did not occur [74]. However, lepirudin was withdrawn from the market in the United States and Europe in 2012 [75].

### 3.4. Direct Factor-Xa Inhibitors

Direct factor-Xa inhibitors block factor Xa activity independently of antithrombin. Several agents can be administered parenterally, including fondaparinux (subcutaneous) and danaparoid (subcutaneously or intravenously). Both fondaparinux and danaparoid are renally cleared, characterized by long half-lives, can be monitored using calibrated anti-Xa assays, and lack antagonists, making bleeding complications more difficult to manage.

Fondaparinux is an effective anticoagulant in the HIT setting and appears to have a low risk of overall complications [76]. According to the 2018 American Society of Hematology Guidelines for the Management of Venous Thromboembolism: Heparin-Induced Thrombocytopenia, patients with acute HIT complicated by thrombosis should discontinue heparin and initiate anticoagulation with fondaparinux [40]. Both fondaparinux and NOACs are considered appropriate options for anticoagulation in these patients [40]. Little information on the use of Fondaparinux in ECMO has been published (Table 3). In the case report presented by Parlar et al., a patient with high suspicion of HIT during ECMO support was treated successfully and safely by Fondaparinux (1 × 2.5 mg/day, subcutaneous) [77]. In a study by Osawa et al., it was shown that fondaparinux might be a reasonable option for HIT treatment in dialysis patients if it is used with caution when other options are not available [78].

Kutlesa et al. conducted a study including 40 adult patients with severe ARDS (20 with H1N1-induced ARDS), treated with VV- ECMO at a tertiary care hospital [79]. Fondaparinux (2.5 mg daily) was administered to three patients (8%) with positive PF4 antibodies, but all three patients died. Two additional patients died before HIT antibody test results became available. Overall mortality was 15/40 patients (37%) [79].

Kutlesa et al. reported a single-center retrospective study involving 112 adult patients who received VV-ECMO for COVID-19-induced ARDS. HIT occurred in 39% (44/112) of patients during ECMO treatment [80]. In those with confirmed HIT antibodies, off-label anticoagulation with fondaparinux (5 mg daily) was started. The authors observed that the effect of fondaparinux on patient outcomes in this setting remains unclear [80].

Danaparoid is a heparinoid composed of heparin sulfate, dermatan sulfate, and chondroitin sulfate. It inhibits factor Xa indirectly via antithrombin and, to a lesser degree, thrombin [81]. With a predictable pharmacodynamic response, the drug has a half-life of approximately 24 h. It exhibits an anti-factor Xa to anti-factor IIa activity ratio of 28:1, compared with a 1:1 ratio for heparin. Steady state is generally achieved after 4–5 days, with renal excretion accounting for 40–50% of total clearance. Danaparoid does not significantly prolong PT and has minimal impact on aPTT, making these tests unsuitable for monitoring. Instead, monitoring should be performed using a danaparoid-specific anti-Xa assay. Monitoring is mandatory for patients with marked renal dysfunction, extreme body weights, or those developing thromboembolic or bleeding complications while on treatment. There are no specific antidotes available [40,82].

According to the British Committee for Standards in Haematology guidelines, danaparoid is a suitable alternative anticoagulant for patients with HIT when administered at therapeutic doses (1B) [83].

A case report from Bauer et al. reported a successful femoral VA-ECMO management with danaparoid in a patient with severe respiratory failure after massive pulmonary embolism and suspected type II HIT [84]. The initial dose was 400 IU/h for 4 h, followed by 300 IU/h (0.5–0.8 U/mL anti-Xa factor activity goal). The procedure resulted in a successful outcome. Removal of the cannula proceeded without major bleeding, and anti-factor Xa activity stayed within the target therapeutic range [84] (Table 3). No circuit clotting, new thrombotic events, or thrombus extension were observed.

**Table 3:** Summary of detailed information about included studies.

	Author (Year Published)	Study Design	No of Patients	Diagnostic Test of HIT	No. of HIT Patients	Type of ECMO, VA, VV	Type of Primary Anticoagulation	Alternative Anticoagulation	Thrombotic Event	Bleeding Event	Mortality
1	Lüsebrink, E et al., 2023 [16]	Retrospective, single-center	373	Detection anti-PF4/heparin antibodies, SRA, HIPA, and/or platelet aggregation test	13/373 (3.5%)	VA-ECMO in the cardiac intensive care unit	A standardized protocol for anticoagulation was used for all patients with an initial bolus of UFH (5000 IU) and continued IV UFH infusion. Target an aPTT of 60–80 s.	Argatroban	Arterial thrombosis (10% vs. 15%, excluded and confirmed HIT group, *p* = 0.627), venous thrombosis (8% vs. 15%, excluded and confirmed HIT group *p* = 0.586)	28% vs. 31%, *p* > 0.999 in excluded and confirmed HIT group	In-hospital mortality (43% vs. 38% in confirmed HIT and excluded HIT groups, *p* > 0.999), mortality after one month (35% vs. 38%, *p* > 0.999), three months (43% vs. 46%, *p* > 0.999), and twelve months (53% vs. 46%, *p* = 0.938)
2	Lubnow M, et al., 2022 [47]	Retrospective observational single-center study using prospectively collected data from the Regensburg ECMO Registry	507	HIT ELISA, HIPA test	16/507 (3.2%)	VV ECMO therapy for severe respiratory failure and VA ECMO for circulatory failure	UFH is used as the standard of care, with goal aPTT set at 50 s for VV-ECMO and 60 s for VA-ECMO	Argatroban dosing aims for an aPTT of 50 s in HIT-suspected patients and 60 s in those with confirmed HIT	Higher rates of thrombosis in ECMO confirmed HIT	A higher incidence of bleeding in the groups temporarily treated with argatroban	/
3	Mang S, et al., 2022 [52]	Observational study	41	ELISA, HIPA test	1 out of 41	Coronavirus disease 2019 (COVID-19) requiring V-V ECMO	/	Argatroban	/	/	/
4	Arachchillage, et al., 2020 [27]	Single-center, retrospective, observational cohort study	298	ELISA, confirmatory tests such as Hemosil AcuStar HIT-IgG, an automated chemiluminescent immunoassay, or a platelet aggregation assay	19/298	VA-ECMO (11/142, 7.7%) and VV-ECMO (8/156, 5.1%)	A heparin bolus dose is given at cannulation, followed by heparin infusion during ECMO; the target heparin anti-Xa concentration was 0.2–0.3 U/mL for VV-ECMO and 0.3–0.5 U/mL for VA-ECMO	Argatroban, rate of 0.2 μg/kg/min, and adjusted to maintain an aPTT of 48–78 s.	89.5% (17/19)	None of the patients with HIT following VA-ECMO had major bleeding. Major bleeding rates in VA-ECMO and VV-ECMO patients were 27.5% (39/142) and 23.7% (37/156), respectively, with bleeding being more common in patients without HIT (*p* = 0.03).	6/19, 31.6% HIT group vs. 89/279, 32.2% in patients without HIT on ECMO (*p* = 0.79). No difference was observed in the mortality rate in patients on ECMO with HIT 1/2, 50% vs. HITT 5/17, 29.4%; *p* = 0.96
5	Pabst D, et al., 2019 [28]	Single-center retrospective study	455	SRA	14/455	/	Continuous UFH with a goal for an aPTT of 50–60 s	The initial dose of argatroban 2 mcg/kg/min, over 1–3 h at aim to a steady-state aPTT of 50–60 s. The dose was then reduced to 0.5–1 mcg/kg/min to maintain the aPTT in the target range	/	/	Mortality 3/14 (21.4%)
6	Glick D, et al., 2015 [21]	Retrospective	119	Heparin-platelet factor 4 immunoassay, the serotonin release assay	1/119	/	Bolus of UFH at ECMO initiation, followed by a recommended infusion of 7.5 U/kg per hour titrated to maintain an aPTT of 40 to 60 s	Argatroban	/	/	Patients suspected of having HIT—significantly higher in-hospital mortality rates (14/23, 61% vs. 31/96, 32%; *p* = 0.01), reflected the more severe thrombocytopenia in this group, indicating a sicker patient population
7	Arachchillage DJ et al., 2022 [21]	Multicenter observational study	152	ELISA, confirmatory tests such as Hemosil AcuStar HIT-IgG, an automated chemiluminescent immunoassay, or a platelet aggregation assay	16/152	Consecutive patients (≥18 years) with severe COVID-19 who were supported by VV ECMO	UFH with heparin anti-Xa of 0.2–0.3 IU/mL or equivalent aPTT unless they had major bleeding. For patients with thrombosis at the initiation or during ECMO the targets were increased up to anti-Xa of 0.5–0.7 IU/mL or equivalent aPTT at local clinical discretion.	Argatroban	10 out 16 patients (62.5%)	/	3 out of 16 patients
8	Kimmoun A, et al., 2018 [13]	Retrospective study	5797	Positive anti- PF4/heparin antibodies	21/5797	VA-ECMO	UFH	Argatroban in 11/21 confirmed HIT patients (52.4%) and danaparoid in 10/21 patients (47.6%).	7/21 (33.3%) patients—arterial or venous thrombosis	12/21 patients (57.1%)	/
9	Vayne C, et al., 2019 [19]	Observational study	57	ELISA, SRA	2 out 57 patients	VA ECMO for at least 5 days	UFH adjusted to maintain aPTT 1.2 and 1.5 for the first 48 h, then, the heparin dose gradually increased to obtain an aPTT ratio between 1.8 and 2.2	Argatroban			2 out of 2
10	Hanna DJ et al., 2022 [62]	Single-center retrospective study	292	SRA, ELISA	12 patients	VA-ECMO, VV -ECMO	UNH bolus administration of 50–100 units/kg at the time of ECMO cannulation plus heparin infusion to maintain an aPTT of 49–67 s (correlating to an anti-factor Xa level of 0.2–0.5 IU/mL)	Bivalirudin titrated to target an aPTT of 46–65 s	60% (6/12) patients	8 patients (66.7%)—major bleeding, minor bleeding-2 patients (16.7%)	60% (6/12)
11	Giuliano K et al., 2021 [72]	Retrospective cohort study	144	Positive PF4/SRA	13/144 patients (9%)	80.6% VA ECMO	Heparin infusion, with a goal aPTT of 50–65 s	Bivalirudin titrated with an aPTT target range of 50–65 s.	0.25 event/patient in HIT positive, 0.22 event/patient in HIT rule-out, 0.32 event/patient in HIT negative	Gastrointestinal bleeding-HIT patients (0.5/patient); HIT negative 0.07 event /patients	Mortality—similar between patients treated with bivalirudin and heparin (69%) and those anticoagulated with heparin alone (62%), 75% in HIT positive
12	Wood KL, et al., 2020 [85]	Retrospective analysis	203	Positive platelet factor 4 test with an optical density value (OD) value exceeding 1.0, SRA	8/203	VA-ECMO	Heparin monitored with ACT every 6 h with a target of 180 to 220 s or activated partial thromboplastin time (aPTT) target of 54 to 71 s	bivalirudin	No	No	No
13	Sullivan J, et al., 2020 [34]	Single-center, observational, retrospective cohort study	39	Positive anti-PF4 result with optical density (OD) of 0.4 or higher and positive SRA results	2/39 (5.1%)	/	Heparin	Bivalirudin and argatroban	No	No	No
14	Kataria V, et al., 2020 [36]	Retrospective, single-center study	473	ELISA with OD greater than or equal to 1.0.; serotonin release assay (SRA)	9/473 (1.9%)			Fondaparinux, argatroban, or bivalirudin	Clinically significant bleeding, defined as bleeding that caused a hemoglobin drop of 2 g/dL or more, occurred more often in the SRA-positive group (36.8% vs. 5 patients, 55.6%, *p* = 0.282).	Venous thromboembolism events—more frequent in the SRA-negative group (31.5% vs. 2 patients, 22.2%, *p* = 0.99	/
15	Mazzeffi M, et al., 2021 [71]	Observational	20	SRA	2 out 20 (10%)	VA ECMO	Heparin with a goal aPTT between 60 and 80 s	Direct thrombin inhibitors	1 of 2 patients	/	No
16	Sokolovic M, et al., 2016 [14]	Retrospective study of a prospectively collected dataset	96	SRA, ELISA test (anti-PF4/heparin antibodies) OD values of equal or greater than 0.4	8 of 96		UFH—aim for ACT goal of 160–180 s (antiXa 0.3–0.7 IU/mL)	Argatroban and bivalirudin	7 of 8 patients	/	/
17	Kutleša M, et al., 2017 [79]	Single-center retrospective study of prospective database	40	ELISA antibodies PF-4	3 out of 40	VV ECMO	UFH-ACT values targeted at the range between 170–180 s	Fondaparinux (2.5 mg daily)	/	/	/
18	Kutleša M, et al., 2023 [80]	Single-center retrospective study	112	ID-PaGIA Heparin/PF4 Antibody Test; ELISA testing	39% (44/112)	VV ECMO for COVID-19-induced ARDS	UFH-ACT values targeted at the range between 170–180 s	Fondaparinux (5 mg daily)	/	/	/
19	Bauer, C. et al., 2008 [84]	Case report	1			VA ECMO		UFH 400 IU/h for 4 h, then 300 IU/h (0.5–0.8 U/mL anti-Xa factor activity goal)	No	No	No

ECMO, Extracorporeal Membrane Oxygenation; VV-ECMO, Venovenous ECMO; VA-ECMO, Venoarterial ECMO; aPTT, Activated Partial Thromboplastin Time; UFH, Unfractionated heparin; COVID-19, Coronavirus disease 2019; ELISA, Enzyme-linked immunosorbent assay; SRA, Serotonin release assay; HIPA, Heparin-Induced Platelet Activation.

### 3.5. Other Anticoagulation Approaches

Factor XIIa inhibitors are new anticoagulants targeting FXIIa and with the potential to prevent thrombosis without increasing bleeding risk; however, they have been evaluated only in animal models of ECMO [38,86,87].

Nafamostat mesylate (NM) is a synthetic serine-protease inhibitor characterized by a very short half-life (5–8 min). NM exerts its effects on coagulation and platelet activity by blocking activated coagulation factors VIIa, XIIa and Xa, thrombin, and plasmin. It also interferes with fibrinolysis and inhibits proteases such as kallikrein, trypsin, and complement components C1r and C1s. Moreover, NM suppresses tissue factor (factor V)-dependent generation of factor Xa in a concentration-dependent fashion, providing localized anticoagulation within ECMO circuits [88]. It undergoes metabolism in the liver and bloodstream and is eliminated via the kidneys and intestines. The best anticoagulation levels of NM are unknown. In the case of ECMO patients, the administered dose of NM showed considerable variability, with mean values ranging from 0.46 to 0.67 mg/kg/h [88]. Monitoring its effect may be measured with aPTT. Reported adverse effects include electrolyte imbalance, including hyperkalemia and hyponatremia, and agranulocytosis [89]. The risk of adverse reactions appears to correlate with both NM concentration or daily dose and treatment duration. NM lacks a specific antidote.

NM has been employed as an anticoagulant in patients undergoing continuous renal replacement therapy in Japan and Korea [90]. In several Asian countries, its approved indications extend to anticoagulation during disseminated intravascular coagulation, extracorporeal circulation in patients who are at risk of, or already experiencing, active bleeding and in treatment of acute pancreatitis [88,91]. NM has also been employed to reduce hemorrhagic complications in hemodialysis and plasmapheresis [38,86]. However, large-scale studies of NM use in ECMO are still lacking. Guidelines for the optimal use of NM as an anticoagulant during MCS therapy are still lacking.

Nitric oxide (NO) releasing polymers have potential applications as thromboresistant coatings for many blood-contacting biomedical devices, as NO is a well-known inhibitor of platelet adhesion and activation [87]. NO that is exogenously added to extracorporeal circuits along with UFH has been shown to reduce platelet activation and consumption so that interaction between platelets and extracorporeal surfaces can be inhibited. Ideally, the future of anticoagulation for ECLS lies in the developing non-thrombogenic circuit that emulates the natural vascular endothelium [92].

The 2022 ELSO Anticoagulation Guideline provides no specific recommendations regarding anticoagulant selection for either VA- or VV-ECMO in the setting of HIT [87]. Further studies are required to confirm efficacy and safety profile with respect to available agents. Recent studies suggest that bivalirudin and argatroban can be equally safe and possibly more effective than UFH. As the use of ECMO increases, these agents may emerge as a viable primary anticoagulants in patients both with and without UFH-related complications. However, these findings are limited by the nature and quality of the included studies (small samples and their single-center, nonrandomized and retrospective design). Moreover, heterogeneity in outcome reporting, without unique definitions and limited adherence to the ELSO definitions of bleeding and thrombosis, makes drawing conclusions difficult [1].

### 3.6. The Alternative Anticoagulation Methods

Despite significant advances in ECMO circuit technology over recent decades, the need for systemic anticoagulation persists. Heparin-coated circuits are currently considered the standard of care in ECMO support and have been widely adopted in clinical practice [93]. However, surface coatings do not eliminate the risk of thrombotic or hemorrhagic complications, particularly when systemic anticoagulation is reduced [94,95]. Although heparin coatings lower the requirement for systemic anticoagulation, they do not abolish the risk of thrombosis or bleeding [94,95]. Chemically bound heparin does not diffuse into the bloodstream; nonetheless, the use of heparin-coated systems can be problematic in patients with suspected or confirmed HIT [96]. Continued use of a heparin-coated circuit following the discontinuation of UFH therapy may contribute to persistent thrombocytopenia [97]. Some authors therefore advocate exchanging the circuit for a heparin-free system in such cases [98]. Conversely, successful management of HIT has also been reported in patients maintained on heparin-coated ECMO circuits. In a comparative study, Koster et al. observed no increase in immunologic or thrombogenic reactions from heparin–PF4–IgG complexes when using heparin-coated circuits compared with non-coated systems [99].

## 4. Future Development and Outlook

Despite the availability of anticoagulation guidelines, there remains a lack of sufficient evidence to establish clear, standardized recommendations for monitoring and anticoagulation treatment in ECMO patients who are highly suspected of or diagnosed with HIT [12,100].

A key limitation of the available studies lies in the inconsistency of outcome definitions, such as minor/major bleeding or patient- and pump-related thrombosis. For more reliable comparison across studies, standardized definitions for these outcomes are necessary [101].

There are significant differences in the diagnostic processes between centers, including variability in diagnostic criteria, the low sensitivity of certain ELISA anti-PF4/heparin antibody assays and lack of standardization of functional tests [13,83].

Future research should focus on identifying patient- and ECMO-related risk factors and predictors of HIT. Additionally, there is a need to develop protocols and standard operating procedures for HIT screening, as well as to revise criteria for further testing and diagnosis of HIT in ECMO patients [14]. Moreover, research should focus on optimizing anticoagulation protocols, refining monitoring strategies, and developing tailored management approaches for ECMO patients with HIT. Since UFH has been associated with HIT and increased mortality in this setting, several reports have explored the feasibility of providing ECMO support without systemic anticoagulation [102,103,104,105]. However, these findings should be interpreted with caution, as some form of anticoagulation was still utilized in these studies.

Current data on the efficacy of DTIs comes from relatively small studies, predominantly retrospective, characterized by substantial heterogeneity in treatment and outcome reporting. The absence of large-scale, prospective randomized controlled trials makes systematic assessment of adverse events challenging. The main limitations of DTIs include their costs, the absence of a specific reversal agent or antidote, and potential to destabilize pre-existing clots. However, given their short half-life, these disadvantages may be less significant. Further research in this area is anticipated.

Emerging preclinical data indicate that antibodies targeting factors XI and XII could offer improved safety and efficacy [5], though clinical data in humans are currently lacking. Results from an ongoing prospective randomized controlled trial assessing the safety and feasibility of argatroban in ECMO patients are anticipated soon (NCT05226442).

Moreover, controlled trials directly comparing bivalirudin with other anticoagulants in ECMO patients are lacking. To fill this gap, two ongoing studies (NCT 03707418 and NCT 03965208) are evaluating the safety and efficacy of bivalirudin compared to UFH in adult ECMO patients [53]; their findings are awaited.

Despite significant advancements in ECMO circuit technology over the past decades, systemic anticoagulation remains necessary. Outcomes in patients receiving ECMO support are influenced by underlying disease, comorbidities, the type of technology used, and anticoagulation management [5].

Significant research is underway to develop new artificial surfaces, with a focus on using synthetic and natural polymers for surface coatings or endothelialization. Despite these advancements, no non-heparin coatings have proven superior to heparin coatings. Heparin continues to be the most widely used option in clinical practice [1].

Given the wide field of indications for ECMO therapy, the number of HIT patients and the challenges involved in designing prospective studies for HIT, creating an international registry could help facilitate clinician reporting and capture real-world outcomes for acute HIT treated with alternative anticoagulant therapy.

## 5. Conclusions

In this contemporary review on HIT management during ECMO, we highlight recent developments and ongoing challenges in therapeutic strategies. Thrombocytopenia and platelet dysfunction in ECMO patients are multifocal, and our understanding remains limited. HIT, while uncommon, represents a serious complication that demands early detection and management to reduce morbidity and mortality. Alternative anticoagulants should be initiated when clinical suspicion is supported by a positive ELISA result. Growing and expanding clinical experience with the DTIs as first-line alternatives to UFH marks a promising direction for the coming years.

## Figures and Tables

**Table 1:** Overview of anticoagulation agents and methods for monitoring the hemostatic system during ECMO.

Anticoagulation Agent	Inhibition Site	Monitoring	Onset/Half-Life	Usual Dose	Elimination Routes
Unfractionated Heparin	Factor Xa and thrombin inhibition, predominantly inactivating thrombin	Anti-FXa, ACT, aPTT, TT	Half-life: 60–90 min	Bolus 50–100 IU/kg, continious infusion 10.4–21.3 IU/kg/h to achieve anticoagulation targets aPTT 40–80 s	Reticuloendothelial system and the kidneys
Nafamostat mesylate	Serine protease inhibitor	ACT, aPTT	5–8 min	1.0–1.7 mg/kg/hr	Metabolism in the liver and bloodstream, eliminated via the kidneys and intestines
Bivalirudin	Direct thrombin inhibitor	ACT, aPPT, PTT, TT, dTT, ECT, Viscoelastic methods, CAA	Half-life: 25 min/ Onset of action 2–4 min	A loading dose ranging from 0.2 to 0.75 mg/kg; maintenance infusion rates ranging from 0.05 to 0.15 mg/kg/h	Metabolism: proteolytic degeneration and partial renal excretion
Argatroban	Direct thrombin inhibitor	ACT, aPTT, drug concentartion, TT, dTT, ECT, Viscoelastic methods, CAA	Half-life: 45 min/Onset of action 30 min	The initial infusion rate 0.2–2 mcg/kg/min; Maintenance dose 0.1–0.2 mcg/kg/min	Metabolism: Liver-dependent
Low-molecular-weight heparin	Factor Ila and Xa inhibition, predominantly inactivating factor Xa	Anti-FXa, aPTT	Half-life: 3–6 h	Enoxaparin, a bolus dose IV 0.5 mg/kg before ECMO cannulation, followed by continuous administration, with anti-Xa target levels of 0.4–0.6 IU/mL	Renal
Lepirudin	Direct thrombin inhibitor	ACT, aPTT, ECT	Half-life: 1–2 h	Bolus of 0.4 mg/kg followed by 0.15 mg/kg/h	Renal
Fondaparinux	Direct-Xa inhibitor	Anti-FXa	Half-life: 13–21 h	1 × 2.5 mg/day	Renal
Danaparoid	Factor Xa and IIa inhibition	Anti-FXa	Half-life: 25 h	400 IU/h for 4 h, then 300 IU/h (0.5–0.8 U/mL anti-Xa factor activity goal)	Renal

ACT, activated clotting time; aPTT, Activated Partial Thromboplastin Time; dTT, Diluted Thrombin time; ECT, Ecarin clotting time; CAA, Chromogenic antithrombin activity.

**Table 2:** The distinction between univalent and bivalent thrombin inhibitors.

Agent	Binding Mode	Sites of Binding	Notes
Argatroban	Univalent DTI	Active (catalytic) site only	Simple active-site blockade
Bivalirudin	Bivalent DTI	Active site + Exosite 1 (fibrinogen-binding)	Initial dual-site blockade, then cleavage at active site, partial binding remains at exosite 1

DTI, direct thrombin inhibitor.

## Data Availability

No new data were created or analyzed in this study. Data sharing is not applicable to this article.

## Abbreviations

The following abbreviations are used in this manuscript:

ECMO	Extracorporeal Membrane Oxygenation
CPR	Cardiopulmonary Resuscitation
VV-ECMO	Venovenous ECMO
VA-ECMO	Venoarterial ECMO
DIC	Disseminated Intravascular Coagulation
SAPS III	Simplified Acute Physiology Score
aPTT	Activated Partial Thromboplastin Time
ICU	Intensive Care Unit
UFH	Unfractionated heparin
AT	Antithrombin
ACT	Activated Clotting Time
HIT	Heparin-Induced Thrombocytopenia
PF4	Platelet Factor 4
HITT	Heparin-Induced Thrombocytopenia with Thrombosis
CPB	Cardiopulmonary Bypass
MCS	Mechanical Circulatory Support
HEP	HIT Expert Probability
LLL	Lilo-Le Louet score
ELISA	Enzyme-linked immunosorbent assay
C-SRA	C-serotonin release assay
HIPA	Heparin-Induced Platelet Activation
DTI	Direct Thrombin Inhibitor
dTT	Diluted Thrombin Time
ECT	Ecarin Clotting Time
CAA	Chromogenic Antithrombin Activity
VKA	Vitamin K Antagonist
BMI	Body Mass Index
LV	Left Ventricle
ICH	Intracranial Hemorrhage
CRRT	Continuous Renal Replacement Therapy
ARDS	Acute Respiratory Distress Syndrome
PT	Prothrombin Time
NM	Nafamostat mesylate
NO	Nitric Oxide
ELSO	Extracorporeal Life Support Organization
